# Identifying Exercise Interventions That May Slow the Progression of Cognitive Decline in Older Adults With Mild Cognitive Impairment: A Scoping Review

**DOI:** 10.7759/cureus.80895

**Published:** 2025-03-20

**Authors:** Kerstin Castro, Jo Ann M Bamdas, Maria D Ortega, Mario Jacomino

**Affiliations:** 1 Medical School, Edward Via College of Osteopathic Medicine, Spartanburg, USA; 2 Medical School, Florida Atlantic University Charles E. Schmidt College of Medicine, Boca Raton, USA; 3 Louis and Anne Green Memory and Wellness Center, Christine E. Lynn College of Nursing, Florida Atlantic University, Boca Raton, USA; 4 School of Nursing and Health Studies, University of Miami, Coral Gables, USA; 5 Women’s and Children’s Health, Florida Atlantic University Charles E. Schmidt College of Medicine, Boca Raton, USA

**Keywords:** alzheimer’s disease, cognitive impairment, exercise, mild cognitive impairment (mci), scoping review

## Abstract

Mild cognitive impairment (MCI) is defined as the stage between normal cognitive changes that may occur with age and more serious symptoms that indicate dementia. Symptoms of MCI can include problems with thinking, judgment, memory, and language, but the loss does not significantly interfere with the ability to handle everyday activities. A wide range of evidence demonstrates that aerobic, strength training, and combined exercises can slow the progression of cognitive impairment. However, few research studies in the United States exist, possibly due to an over-reliance on pharmacological intervention. To answer our research question, we performed a scoping review analysis of randomized controlled trials (RCTs) to locate published studies showing the types of exercise interventions using cognitive measures that benefit communities of older adults. Using Preferred Reporting Items for Systematic Reviews and Meta-Analyses extension Scoping Reviews guidelines, the authors searched five databases, namely, PubMed, GALE, Wiley Online Library, EBSCOHost, and OVID, for exercise intervention programs and slowing the progression of cognitive decline in older adults with MCI. All searches were performed between September 2022 and February 2023. In summary, we analyzed 10 international RCTs with 1,075 participants (mean age of over 50 years), but only 668 people were included in the control or experimental group. Combining exercise modalities in the intervention groups versus control groups prompted more beneficial results, improving global cognitive functioning. Resistance training shows promise as an exercise modality suitable for older adults to slow the progression of cognitive impairment.

## Introduction and background

Mild cognitive impairment (MCI) is defined by the National Institute of Neurological Disorders and Stroke (NINDS) as the stage between normal cognitive changes that may occur with age and more serious symptoms that indicate dementia. Symptoms of MCI can include problems with thinking, judgment, memory, and language, but the loss does not significantly interfere with the ability to handle everyday activities [1]. Up to 19% of those aged over 65 years are affected [2]. The criteria for diagnosing MCI include a subjective concern for change in cognition, impairment in one or more cognitive domains, preservation in independence of functional abilities using clinical and cognitive evaluation, and the absence of dementia [3]. Some people with MCI seem to remain stable or return to normal over time, but more than half progress to dementia within five years [2]. MCI is differentiated from Alzheimer’s disease via intact activities of daily living and the absence of dementia [4]. The high percentage of 19% described earlier is cause for concern due to the growing population of older adults aged over 65 years in the United States. Currently, 9% of the population is 65 years and older, which is expected to rise to 17% by 2050 [5]. Using the projections from the 2020 Census, the percentages quoted imply that about 117 million people in the United States have MCI, which will likely increase to 304 million by 2050 [5]. These findings lead to a need for more caretakers or other healthcare providers, for which there is already a shortage [6].

Older adults with MCI face an increased risk of progressing to dementia, particularly Alzheimer’s disease. Because other cognitive domains can be impaired in individuals with MCI, it is essential to examine domains besides memory, including executive functions, language, visuospatial skills, and attention control [3]. As a result, the detrimental consequences of cognitive decline in this population include impaired executive function, difficulty maintaining independence, increased risk of falls and injuries, and a more significant burden on caregivers. Additionally, cognitive deterioration can lead to social withdrawal, depression, and a reduced quality of life. Some conditions that may indirectly lead to cognitive decline can be reversible or irreversible. Reversible causes may be neurological disorders (e.g., normal-pressure hydrocephalus), metabolic disorders (e.g., hypothyroidism, hypoglycemia, hyperglycemia, dehydration, and vitamin B12 deficiency), depression, polypharmacy, atrial fibrillation, hypotension, or hypertension [7]. These reversible causes are the targets of treatment interventions. Exercise is currently being recommended as a possible treatment for some of these reversible causes.

A wide range of evidence [6,7] demonstrates that physical activity with aerobic, strength training, and combined exercises may slow the progression of cognitive impairment. Hence, these can be incorporated into post-discharge care plans for patients with MCI. A literature review examined how exercise can be prescribed as a non-pharmacologic intervention for 26 diseases, including neurological diseases such as dementia [8]. In this review, the authors found that a possible mechanism of action for how exercise treats neurological disorders such as dementia was the effect of exercise on the hippocampus. The review claimed that exercise increases adult hippocampal neurogenesis, increases hippocampus volume, and improves spatial learning ability [8]. The review also reported that regular physical activity induces anti-inflammatory effects, which may contribute to explaining the positive effects of exercise in treating dementia [8]. As mentioned, MCI lies between normal cognition and dementia. Therefore, exercise can be used as a non-pharmacologic intervention to prevent or slow down the progression of cognitive decline.

The purpose of this scoping review is to identify the types of exercise interventions showing positive effects in slowing the progression of cognitive decline in older adults with MCI living in various community settings. A lack of research in the United States points to a gap in analyzing the effects of exercise in slowing cognitive decline in individuals with MCI. The significance of this scoping review is to highlight international studies being performed that may illuminate exercise variety that may contribute to slowing the progression of MCI.

## Review

Methodology

We conducted a scoping review to analyze international literature between September 2022 and February 2023 to summarize and disseminate areas identified as not being addressed by researchers in the United States. According to Armstrong et al., a scoping review would likely reveal numerous interventions used in various settings and help identify a more specific research question of interest [9]. Our methodology/approach for this study follows the Joanna Briggs Institute (now JBI) guidelines for scoping reviews [10]. It involves a detailed and organized literature review using a conceptual framework to identify relevant articles, followed by data extraction and analysis. Peer-reviewed studies addressing cognitive impairment (decline) with aerobic, strength training, and combined exercise programs were included. Broad terms were incorporated into the search of our literature review related to cognitive impairment or cognitive decline, exercise programs, transitions of care, and hospital admission of older adults. We relied on identifying a research question with relevant studies, selecting studies, charting the data, summarizing, and reporting the results.

Our research question focused on what types of exercise intervention programs are effective in slowing the progression of cognitive decline in older adults with MCI? To answer this question, we obtained international articles in English and Spanish for this review from five databases, namely, PubMed, GALE, Wiley Online Library, EBSCOHost, and OVID. All searches were performed between September 2022 and February 2023, resulting in 420 articles.

**Figure 1:**
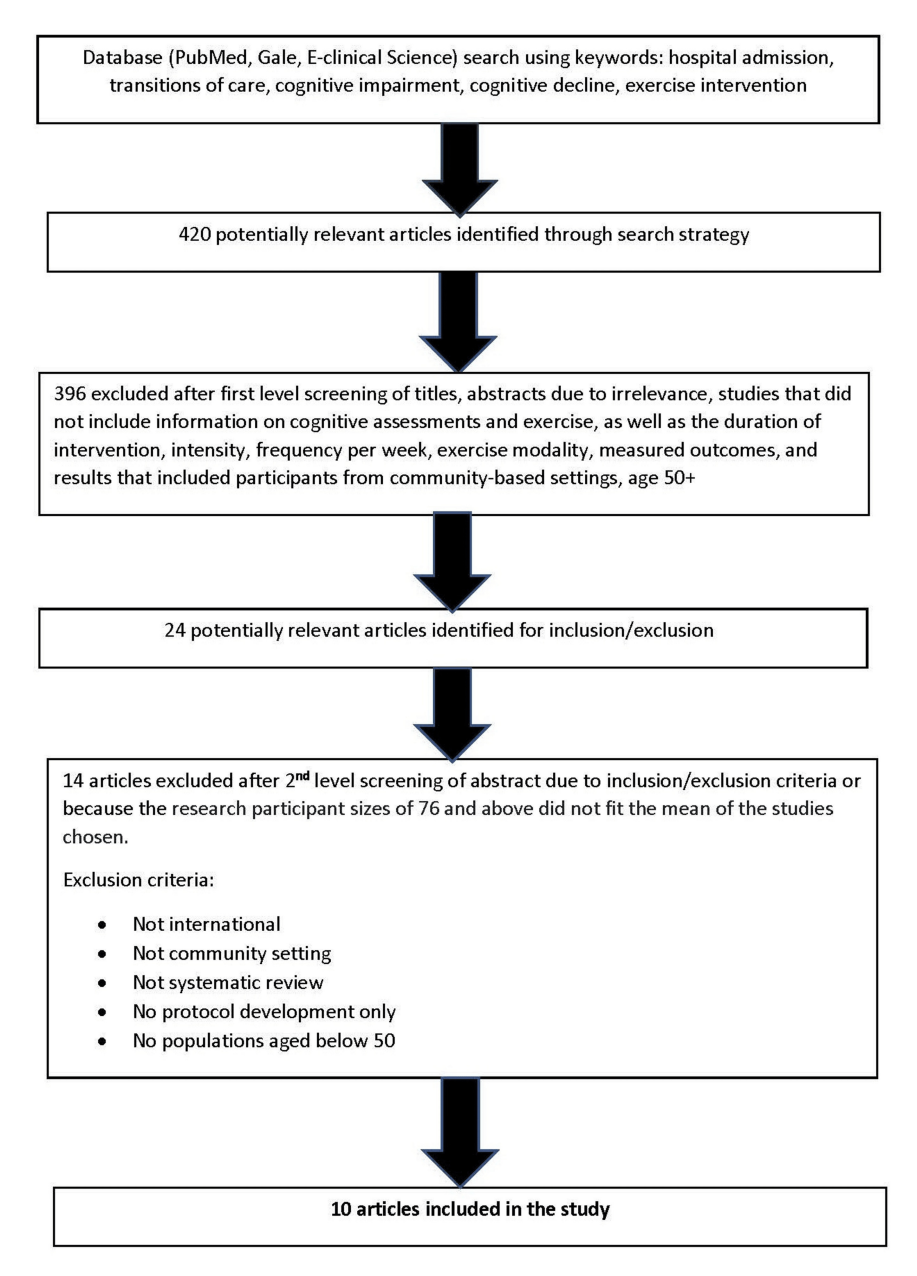
Flow diagram of articles meeting the search criteria.

The first author screened these articles to determine whether they were unique (i.e., not appearing in multiple sources) and as a primary study for exercise intervention. During this first-level screening, we also eliminated studies that did not include information on cognitive assessments and exercise, as well as the duration of intervention, intensity, frequency per week, exercise modality, measured outcomes, and results that included participants from community-based settings aged 50+ years. A second-level screening was done to exclude randomized controlled trials (RCT) that were not international, not set in a community setting, systematic review, protocol development only, and populations aged below 50 years. RCTs with research control or experimental group participant sizes of 76 and above did not fit the mean of the studies chosen for our review and were excluded from this study. In summary, we analyzed 10 international RCTs, as shown in Figure 1, with 1,075 participants (mean age of over 50 years), but only 668 participants were included in the control or experimental group.

Data analysis

We identified 10 studies with a randomized control design. RCTs have been described as a gold standard to measure an intervention’s effect by randomly assigning two or more individuals to groups to test a specific treatment, drug, or other intervention, such as exercise programs [11]. Each reviewed article presented results showing certain exercises slowing the progression of MCI. Results were examined and cataloged if a significant change (increase or decrease) emerged between baseline cognitive and post-exercise intervention assessments. Potentially relevant RCTs with research participant sizes of 76 and above did not fit the mean of the 10 studies chosen for our review, so they were excluded from this review.

Generalization, Reliability, Validity, and Bias

Data analysis focused on the Population, Intervention, Control, and Outcome (PICO) format. The authors concluded that the overarching research question explored was feasible for the relevant populations examined in the selected RCTs. Moreover, the interventions were reproducible. The study size for this review was 357 in the experimental group and 311 in the control group. The control groups were specific, and the outcomes were relevant to experimental evidence, ethical methods, and procedures. As researchers, we analyzed the p-value, which does not always indicate significance, no difference, or always more significance. We examined the test scores and the p-values with an expert in neuropsychological testing and discussed every aspect of the studies under study. After reviewing the RCTs, we created a thematic analysis of coded repeated words and/or phrases.

Results

Out of 420 potentially relevant articles identified through the search strategy, 396 were excluded after the first-level screening of titles and abstracts. During this first-level screening, we also eliminated studies that did not include information on cognitive assessments and exercise, as well as the duration of intervention, intensity, frequency per week, exercise modality, measured outcomes, and results that included participants from community-based settings, aged 50+ years. The first author identified 24 potentially relevant articles for inclusion or exclusion. After the second-level screening, 12 articles were excluded due to the exclusion criteria, i.e., not international, not community setting, not an RCT, no systematic reviews, no protocol development, and no populations aged below 50 years. Two additional studies were excluded because the research participant sizes of 76 and above did not fit the mean of the other chosen studies for our review. Finally, 10 articles were included in the analysis. While sex/gender was initially one of the criteria, most research studies sampled “community-dwelling adults” rather than randomizing males or females into a control group or intervention group.

Most studies that directly examined the effects of exercise intervention in older adults diagnosed with MCI were international studies by international researchers. Seemingly, this indicated that it is possible that researchers in the United States are not addressing this population, particularly using RCTs or focusing on other challenges such as research and development of drugs and/or specific sections of the brain.

Furthermore, each study’s results depend on the participant’s social norms, which vary by country. Other countries have different social norms about physical activity and diet, so the same study in the United States could have completely different results. In China, 66.3% of adults are physically active, while in the United States, 24.2% meet physical activity guidelines [12].

Table 1 summarizes the studies analyzed in an adapted PICO format, as indicated in the results and discussion. We also calculated the increased or decreased percentages of the cognitive status scales included in each article examined. We calculated these using a simple percent increase and decrease formula (new - old)/old. For each study, we used the pre-intervention test results (old) and the post-intervention test results (new). The purpose of this was to determine if there were any notable changes between test results for each exercise intervention, regardless of whether the p-values were statistically significant or not, to evaluate if there was any improvement. This might point to a potential benefit of certain exercise interventions in slowing the progression of MCI, but most of the studies had time limitations that prevented the actual statistical significance. This is shown in the last column of Table 1.

**Table 1:** Summary of studies referenced assessing exercise interventions, testing methods, and results for comparison analysis. Exp.: experimental; HRR: heart rate reserve; RM: repetition maximum; CMMSE: Categorical Mini-Mental State Examination; IADL-C: Instrumental Activities of Daily Living – Cognitive; DST-B: Digit Symbol Test-Backward; DST-F: Digit Symbol Test-Forward; ADAS-Cog: Alzheimer’s Disease Assessment Scale-Cognition; TMT-B: Trail Making Test B; CWF: Categorical Word Frequency; WMS-LM: Wechsler Memory Scale – Logical Memory; MoCA: Montreal Cognitive Assessment; SDMT: Symbol Digital Modalities Test; RAVLT: Rey Auditory Verbal Learning Test; WAIS: Wechsler Adult Intelligence Scale; LM II: Logical Memory Test II; BDS: Brief Dementia Scale; ANOVA: analysis of variance; RM ANOVA: repeated-measures analysis of variance; MMRM: mixed models for repeated measures; ANCOVA: analysis of covariance

Study	Sample experiment	Sample control	Length (weeks)	Exercise modality	Exercise intensity	Frequency/week	Primary cognitive assessment (multiples)	Test statistics	P-value	% Increase/Decrease
Siu and Lee, 2018 [13]	74	71	16	Tai Chi	N/A	2X	CMMSE	Two-tailed t-tests, Fisher’s exact test, chi-square	0.001	5.03%↑
Siu and Lee, 2018 [13]	74	71	16	Tai Chi	N/A	2X	IADL-C	Two-tailed t-test, Fisher’s exact test, chi-square test	0.004	3.40%↑
Hong et al., 2018 [14]	10	12	12	Resistance band	15RM	2X	DST-F	ANOVA, t-test	0.032	0%↑
Lü et al., 2015 [15]	22	23	12	Dumbbell	1.92kg	3X	ADAS-Cog (C)	ANOVA, chi-square test	0.012	-23.3%↓
Lü et al., 2015 [15]	22	23	12	Dumbbell	1.92kg	3X	TMT-B (C)	ANOVA, chi-square test	0.003	-25.9%↓
Lü et al., 2015 [15]	22	23	12	Dumbbell	1.92 kg	3X	DST-B (C)	ANOVA, chi-square test	0.025	12.0%↑
Lü et al., 2015 [15]	22	23	12	Dumbbell	1.92 kg	3X	DST-F (C)	ANOVA, chi-square test	0.579	1.93%↑
Varela et al., 2011 [16]	27	15	24	Cycling	40%HRR	3X	MMSE (S)	ANOVA, Newman-Keuls test	0.776	4.02%↑
Varela et al., 2011 [16]	26	N/A	24	Cycling	60%/HRR	3X	MMSE (S)	ANOVA, Newman-Keuls test	0.776	1.20%↑*
Maki et al., 2012 [17]	75	75	12	Walking	N/A	1X	CWF	RM ANCOVA, t-tests	0.01	7.50%↑
Zhu et al., 2018 [18]	29	31	12	Dance	60-80% max HR	3X	WMS-LM	MMRM	<0.05	16.8%↑
Zhu et al., 2018 [18]	29	31	12	Dance	60-80% max HR	3X	MoCA (B)	MMRM	<0.001	7.76%↑
Zhu et al., 2018 [18]	29	31	12	Dance	60-80% max HR	3X	TMT-A	MMRM	<0.05	-18.9↓
Zhu et al., 2018 [18]	29	31	12	Dance	60-80% max HR	3X	SDMT	MMRM	<0.05	15.6%↑
Nagamatsu et al., 2013 [19]	28	N/A	24	Resistance	7 RM	2X	RAVLT	Univariate ANOVA, RM ANOVA, bivariate Pearson	≤0.05	8.51%↑
Nagamatsu et al., 2013 [19]	30	N/A	24	Walking (outdoor) aerobic	40%, 70-80% HR	2X	RAVLT	Univariate ANOVA, RM ANOVA, bivariate Pearson	≤0.05	9.60%↑
Nagamatsu et al., 2013 [19]	N/A	28	24	Balance and tone training	N/A	2X	RAVLT	Univariate ANOVA, RM ANOVA, bivariate Pearson	≤0.05	6.71%↑
Singh et al., 2014 [20]	27	27	26	Combined	80-92% 1 RM	2X	ADAS-Cog	MMRM, z-scores	<0.005	-43.5%↓
Singh et al., 2014 [20]	22	N/A	26	Resistance bands	N/A	2X	WAIS	MMRM, z-scores	<0.02	6.95%↑
Sungkarat et al., 2017 [21]	33	33	15	Tai Chi	N/A	3X	LM-II	Shapiro-Wilk test, t-test, chi-square test	0.006	46.5%↑
Sungkarat et al., 2017 [21]	33	33	15	Tai Chi	N/A	3X	BDS	Shapiro-Wilk test, t-test, chi-square test	0.01	18.6%↑
Sungkarat et al., 2017 [21]	33	33	15	Tai Chi	N/A	3X	TMT-BA	Shapiro-Wilk test, t-test, chi-square test	0.005	-29.2%↓
Wei et al., 2014 [22]	30	24	12	Handball	60% max HR	5X	MMSE	Chi-square test, t-test, RM ANOVA	0.024	5.06%↑

Table 2 identifies the cognitive assessments from each study included in this scoping review.

**Table 2:** Summary of cognitive assessments used in the studies included in the scoping review including acronyms/abbreviation, name of the assessment, and function evaluated.

Acronym	Name of the cognitive assessment	Function evaluated by the assessment
ADAS-Cog	Alzheimer’s Disease Assessment Scale-Cognition	Global cognitive function
BDS	Brief Dementia Scale	Recall
C-MMSE	Categorical Mini-Mental State Examination	Impairment severity
CWF	Categorical Word Frequency	Word fluency/frontal lobe, visuospatial ability, sustained attention
DSB	Digit Span Backwards	Working memory
DST-B	Digit Symbol Test-Backward	Immediate memory/attention
DST-F	Digit Symbol Test-Forward	Processing speed, visual scanning, motor speed, short-term visual memory, and attention/concentration
IADL-C	Instrumental Activities of Daily Living – Cognitive	Activities of daily living
LM-II	Logical Memory Test II	Logical memory, movement
MMSE	Mini-Mental State Examination	Impairment severity
MoCA	Montreal Cognitive Assessment	Attention, language, function
RAVLT	Rey Auditory Verbal Learning Test	Improved spatial performing
SMT	Snellgrove Maze Task	Attention, visuo-constructional skills, executive functions of planning and foresight
TMT B-A	Trail Making Test B-A	Task switching
TMT-A	Trail Making Test A	Visual perception, speed
TMT-B	Trail Making Test B	Executive function
WAIS-M	Wechsler Adult Intelligence Scale – Memory	Global cognitive function
WMS-LM	Wechsler Memory Scale – Logical Memory	Memory, processing speed

Siu and Lee [13] performed a study examining the effects of a Tai Chi intervention in older adults with MCI in China. In the United States, Tai Chi is not a part of the culture; perhaps it would be more difficult for participants to understand and complete the intervention. Five studies were performed in China. Of the five studies, two used Tai Chi as the exercise intervention. Because many U.S. adults continue to be less physically active and, in many cases, sedentary, an exercise intervention program might have more exaggerated results than the Chinese studies examined. Hong et al. performed their study in Korea [14], where physical activity among adults is decreasing, which could be more indicative of what the results would be like compared to the United States.

Resistance Exercise Intervention Group

Resistance exercise interventions from two research teams (Hong et al. [14] and Lü et al. [15]) included varying modalities such as elastic bands, dumbbells, and functional training sports (squats). Resistance exercise appears to have a more prolonged effect, with improvements lasting for months past the end of the interventions.

Elastic resistance bands were used in one intervention [14], with a positive result (p = 0.032) on the Digit Span Backwards Test cognitive test that tests working memory. There was no increase or decrease.

Dumbbells were used in Lü et al.’s [15] intervention group using four cognitive tests. However, the intervention protocol yielded improvements in only two different cognitive assessments. The first assessment was a p-value of 0.012 for the Alzheimer’s Disease Assessment Scale-Cognitive Subscale (ADAS Cog), a 23.3% decrease, showing global cognitive function. With this cognitive subscale, a decrease in score indicates improvement. The second test was the Trail Making Test Part B (TMT-B), a 25.9% decrease, which assesses executive functioning. The p-value of 0.003, a lower score, indicates an improvement toward the lack of cognitive decline. The cognitive assessment of Digit Symbol Test-Backward (DST-B) showed a p-value of 0.025 and a 12.0% increase, which assesses immediate memory and attention. The final cognitive assessment, Digit Symbol Test-Forward (DST-F), showed a p-value of 0.579 and a 1.93% increase. With this assessment, a higher score is desirable.

Aerobic Exercise Intervention Group

The protocol for the intervention group (Varela et al. [16], Maki et al. [17], Zhu et al. [18]) provided research participant autonomy. For example, participants could choose to jog or walk. Aerobic exercise interventions included cycling on recumbent bikes, walking, jogging, and dancing from three international studies using an RCT control group and an intervention group. Although aerobic exercise showed significant improvements, it did not have lasting effects, diminishing shortly after stopping the intervention.

Cycling and Recumbent Bikes

In the study by Varela et al. [16], the Mini-Mental Status Exam (MMSE) score showed a p-value of 0.776, a 4.02% and a 1.20% increase, significantly increasing or decreasing the score. This score indicates a lack of significance in what they were testing, differences in intensity rather than improvement in the score. However, the researchers concluded that the difference in intensity was not great enough to find significant results. The team utilized a recumbent bike as the aerobic exercise intervention and found that the intervention did not significantly improve MMSE scores.

Walking or Jogging

Maki et al. [17] showed a significant improvement in the Categorical Word Fluency test (p =0 .01), with a 7.50% increase, which is what the researchers hoped for in the intervention. This test assesses word fluency, visuospatial ability, and sustained attention. The researchers created a walking program for the exercise intervention and found that word fluency and functional capacity significantly improved.

Dancing

Zhu et al. [18] provided a standardized dance routine consisting of a specially designed dance routine involving cognitive effort for patients to memorize the complex movements. A dance instructor taught them the routine, and after three months, the intervention group participants could do the routine at home. The movements consisted of knee bending, heel up, boxing, shoulder movements, kicking, square stepping, and sculling exercises. Four cognitive assessments were given, each showing significant improvement. Three showed increases, and the fourth showed a decrease. The tests were as follows: the Weschler Memory Scale Revised Logical Memory tested memory and processing speed with a p-value <0.05, with a 16.8% increase, which is significant. The second test was the Montreal Cognitive Assessment (MoCA), which tests attention, language, and function, resulting in a p-value <0.001, with a 7.76% increase, which proved significant. The third was TMT-A, which tests visual perception and speed, in which the intervention group had a p-value <0.05 and an 18.9% decrease, which is desired for significance. Finally, the fourth test 4 was the Symbol Digital Modalities Test, which tested cognitive function over time, with a p-value <0.05 and an increase of 15.6%, which was significant. This research group created a dance program for the aerobic intervention and found that memory, processing speed, and cognitive function improved significantly, as well as a greater improvement in episodic memory.

Combined Exercise Intervention Group

In five RCTs (Nagamatsu et al. [19], Singh et al. [20], Siu and Lee [13], Sungkarat et al. [21], Wei and Ji [22]), exercise interventions combined resistance and aerobic training. The interventions consisted of handball and Tai Chi. Handball is a combined exercise that consists of toss training, hit training, bounce training, pass training, grab training, field going training, roll training, and pinch training. These trainings benefit the heart (intensity) and exercise the muscles that qualify as resistance. Both exercises improve gait balance.

Multiple Cognitive Tests

Four used numerous cognitive tests, but for this exploration of RCTs, the following tests were found to be significant. Nagamatsu et al. [19] performed the Rey Auditory Verbal Learning Test test for resistance exercises that resulted in a p-value of 0.05, while Singh et al. [20] performed ADAS-Cog testing global testing function that resulted in a p-value <0.05, with a 43.5% decrease, indicating a score that revealed cognition improvement after the training intervention. Also included in the Singh et al. [19] study was the Weschler Adult Intelligence Scale (WAIS-M) test for examining memory that resulted in a p-value <0.02, a 6.95% indicating an increase in the score, alerting to improved cognition.

Siu and Lee [13] utilized the C-MMSE to score impairment severity. The study’s intervention group explored Tai Chi. The significant improvement showed a p-value of 0.001, with an increase of 5.03%. The other test, Instrumental Activities of Daily Living - Cognitive (IADL-CV), showed a 3.40% increase and a p-value of 0.004.

Sungkarat et al. [21] used three types of assessments for the study. The assessments were LM-II, Block Design Subset (BDS), and TMT-B-A. All tests were significant. Two tests (LM-II and BDS) showed an increase (46.5%, p = 0.006, and 18.6%, p = 0.01, respectively), and one test (TMT-B-A) showed a decrease of 29.2% (p = 0.005).

Wei et al. [22] utilized one assessment (MMSE) with handball exercise that signaled a 5.06% increase (p = 0.024), showing a significant decrease in impairment severity in cognition.

Thematic Analysis

In addition to analyzing the percentage increase and decrease of each activity within the intervention group, we conducted a thematic analysis of repeated ideas that emerged during examination across studies and groups, including memory/cognition tests and exercise interventions. After analyzing the 10 articles, two major themes emerged from the effectiveness of the exercise intervention, namely, adherence and intensity. Most researchers measured adherence as the percentage of weekly participation, but some researchers defined adherence as “compliance.”

Theme 1: Adherence

Adherence emerged in a thematic analysis of the trials. For example, Lu et al. [14] described adherence as “compliance” and defined a good attendance rate as an average of 31 sessions. This study outcome had a low attrition rate and satisfactory compliance, suggesting that dumbbell-spinning exercises are a reasonable intervention modality for older adults with MCI.

Nagamatsu et al. [19] also described adherence as “compliance” and measured the percentage of total attendance. The researchers measured 54% exercise compliance for the resistance training group, while the aerobic training group had 60% compliance, which is considered low regarding adherence to the intervention. As this study had a lower compliance rate with the intervention, it suggests that the improvements in cognition are estimates for the effects of resistance training on older adults with MCI. The lower compliance rate could also demonstrate that the required modalities are not best for this population.

Siu and Lee [13] attempted to maximize adherence by requiring the participants to sign attendance records, having an average attendance rate of 81.4%. Still, they did not discuss its possible connection to the program’s effectiveness. As the attendance rate was above 80%, Tai Chi could be a favorable exercise intervention for older adults with MCI. Siu and Lee [13] reported that the main reasons the participants missed the Tai Chi classes were “not feeling well,” poor weather conditions, or attending medical follow-up. As poor weather conditions caused some participants to miss Tai Chi class, perhaps an interchangeable indoor and outdoor setting would increase the adherence even further.

Theme 2: Intensity

An additional theme to emerge from the analysis identified intensity as to how difficult or how much strain the participant experiences during the intervention. Each study indicated a different intensity, suggesting that intensity is associated with positive outcomes from exercise intervention.

Hong et al. [14] measured intensity individually for each participant using their 15-repetition maximum (RM), or 65% of their 1 RM. The study found that using a 15 RM intensity when using elastic resistance bands as a modality successfully improved electroencephalogram patterns in older adults with MCI. Lü et al. [15] used a consistent intensity throughout the study; each participant used a 1.92 kg dumbbell. This study did not discuss the potential benefits of using different intensities.

Nagamatsu et al. [19] allowed participants to choose the free weight of their choice and required them to perform two sets of six to eight repetitions, increasing the load as exercise sessions continued. Singh et al. [20] utilized a high-intensity progressive resistance training program with a set intensity of 80-92% of 1 RM. This study found that high-intensity training had “large significant effects on memory,” while lower-intensity resistance training had inconclusive results. Uffelen et al. [23] individualized the intensity at which each participant would perform the intervention by calculating each subject’s metabolic equivalents.

Varela et al. [16] compared a moderate-intensity walking program, walking at 60% of max heart rate, and a low-intensity walking program, walking at 40% of max heart rate. Varela et al. did not find significant differences in the effects of the different intensity programs. Wei et al. [22] aimed for 60% of the maximum heart rate for the participants. Zhu et al. [17] utilized a 60-80% maximum heart rate. Sungkarat et al. [21] utilized a Tai Chi program, so intensity was not specified.

Discussion

This scoping review provides a broad appraisal and a glimpse into the research question for significant benefits within various international researchers’ exercise programs through exploring RCTs using cognitive tests. Our study shows that specific areas of cognition may improve with certain exercises. Improvement in one area or domain does not necessarily result in improvement in overall cognition. The most important finding of our review shows that there is a level to which the exercise programs were proving beneficial. Researchers using a combination of exercises, namely, resistance training with elastic bands, Tai Chi, and dance, showed significant results on cognitive assessments. Nearly all of these studies showed adherence and intensity emerging as meaningful. Resistance training or a combination of resistance and aerobic-based training was found to have the most significant benefitting patients with MCI in the groups tested.

The implications of our study suggest that a personalized approach to an individual’s exercise plan would be beneficial for slowing the progression of MCI and should include a resistance training modality to maximize benefits. We need to look at each patient as a whole, not as individual components or by cognitive domains. This will help us better identify the causes of our findings. The resistance modality should be appropriate in terms of the physical capabilities of the individual to prevent injury and avoidance due to lack of motivation.

Limitations and future research

MoCA is now seen as the most sensitive assessment for screening and monitoring patients for MCI, so a significant change on that test would hold more than a change on the MMSE or just Trails. Only one RCT included in our study utilized MoCA as the assessment tool. Other assessment tools were used by the studies analyzed in this review. These other assessments do not measure all domains and are not for monitoring the progression or reversal of MCI. Hence, even if a patient does Tai Chi and increases their scores on the MMSE and IADL, they may only improve in one area, which cannot be enough to say they have slowed the progression of MCI.

Although this scoping review examined 10 articles, the results cannot be generalized. This is due to limited research in RCTs testing the efficacy of exercise prescriptions against the progression of cognitive decline in patients with MCI outside of the United States. This scoping review only explored international peer-reviewed articles due to the limited number of RCTs on exercise with cognitive assessments in the United States. Therefore, these topics are ripe for future research. Physical activity, diet, and other social norms in other countries differ from the United States. In addition, the United States concentrates on treating MCI with new pharmacological approaches compared to other global communities. The 10 studies examined did not have uniformity among cognitive assessments. These studies also had a wide range of improvements with different tests. In addition, most RCTs had time limitations that prevented the actual statistical significance.

This research followed a rigorous methodology, clear research questions, statistically significant results, and replicability. The results can be verified by other researchers. It is an initial exploration of cognitive assessments being used in RCTs to determine the significance of the exercise that may slow the progression or even reverse MCI in older adults. A future study should focus on a single exercise intervention via an RCT. In addition, future studies must have a universal cognitive assessment tool to measure outcomes, making MoCA the ideal tool. When examining older adults with MCI, exercise intervention modalities must be within the range of physical and medical capabilities of the particular group. Additional students could examine adherence more closely, as it is a factor in the effectiveness of exercise intervention.

## Conclusions

A wide range of evidence indicates that exercise can slow the progression of cognitive decline in older adults with MCI. As the studies included in this scoping review were international, it is important to bring the information to the United States. However, a possible confounding variable is the societal norms that dictate a person’s diet and physical activity levels. The United States treats MCI with new pharmacological approaches compared to other global communities. For this reason, there is very limited research using controlled studies to test the efficacy of prescribing exercise against the progression of cognitive decline in patients with MCI in the United States. With physical inactivity causing premature death and cognitive decline, finding pleasurable exercises in the United States is more critical than ever. Future studies should evaluate the population’s social norms to understand the various aspects of different individuals better and the notable differences between racial and ethnic groups to understand this variable better. Exercise interventions require adherence to the program, and it is difficult to measure and ensure adherence to an exercise program. In future studies, adherence should be isolated and then examined to determine the extent to which adherence alters exercise intervention program effectiveness. In addition, future studies should be conducted for a longer time as most RCTs that were reviewed had time limitations that could prevent the actual statistical significance.
